# Effectiveness of Dental Restorative Materials in the Atraumatic Treatment of Carious Primary Teeth in Pediatric Dentistry: A Systematic Review

**DOI:** 10.3390/children12040511

**Published:** 2025-04-16

**Authors:** Gianna Dipalma, Angelo Michele Inchingolo, Lucia Casamassima, Paola Nardelli, Danilo Ciccarese, Paolo De Sena, Francesco Inchingolo, Andrea Palermo, Marco Severino, Cinzia Maria Norma Maspero, Alessio Danilo Inchingolo

**Affiliations:** 1 Department of Interdisciplinary Medicine, University of Bari “Aldo Moro”, 70124 Bari, Italy; gianna.dipalma@uniba.it (G.D.); a.inchingolo3@studenti.uniba.it (A.M.I.); lucia.casamassima@uniba.it (L.C.); paola.nardelli@uniba.it (P.N.); danilo.ciccarese@uniba.it (D.C.); p.desena@studenti.uniba.it (P.D.S.); alessiodanilo.inchingolo@uniba.it (A.D.I.); 2 Department of Experimental Medicine, University of Salento, 73100 Lecce, Italy; andrea.palermo@unisalento.it; 3 Department of Medicine and Surgery, University of Perugia, Severi Square n.1, 06132 Perugia, Italy; marco.severino@unipg.it; 4 Department of Biomedical, Surgical and Dental Sciences, School of Dentistry, University of Milan, 20100 Milan, Italy; cinzia.maspero@unimi.it

**Keywords:** dental restorative materials, Atraumatic Restorative Treatment (ART), dental caries, pediatric dentistry

## Abstract

Aim: This systematic review evaluates the effectiveness and clinical outcomes of Atraumatic Restorative Treatment (ART) in pediatric dentistry, comparing it with other restorative techniques, analyzing material performance, assessing cost-effectiveness, and exploring the long-term success in managing dental caries. Background: ART is a minimally invasive approach that removes decayed tissue using hand instruments and restores teeth with adhesive materials like glass ionomer cement (GIC). ART is particularly valuable in pediatric dentistry due to its simplicity, reduced discomfort, and suitability for resource-limited settings. It eliminates the need for anesthesia and expensive dental equipment, making it accessible in remote and underserved areas. Studies have shown its effectiveness in providing durable restorations while improving patient comfort. Materials and Methods: This systematic review follows the PRISMA guidelines. PubMed, Web of Science, and Scopus were searched for studies published in the last ten years. The inclusion criteria included in vivo studies on children, randomized controlled trials, and case–control studies assessing ART’s effectiveness. Quality and risk of bias were evaluated using the ROBINS-I tool. Results: Eighteen studies met the inclusion criteria. ART effectively managed dental caries, especially with high-viscosity GIC. Comparisons with the Hall Technique and Papacarie showed that ART remains a viable, cost-effective option. Conclusions: ART is a reliable, minimally invasive technique for pediatric restorative dentistry. Its accessibility and cost-effectiveness make it suitable for low-resource settings. High-quality materials and technique modifications further enhance restoration longevity.

## 1. Introduction

Atraumatic Restorative Treatment (ART) is an innovative, minimally invasive dental technique designed for treating caries, particularly in pediatric dentistry [1,2,3]. This method relies on the use of manual instruments to remove decayed tissue and the application of adhesive materials such as glass ionomer cement (GIC) to restore the tooth [4,5,6,7,8]. ART stands out for its simplicity and effectiveness, addressing the need for more gentle treatments that minimize pain and discomfort, especially for younger patients [9,10,11].

One of the main advantages of ART is its accessibility, making it particularly suitable for environments with limited resources [12,13,14,15,16]. Unlike traditional techniques, ART does not require expensive equipment or advanced technology, reducing the cost of treatment and making it available even in rural areas and disadvantaged communities, where dental facilities are often lacking [17,18,19]. ART proves to be a highly effective and affordable option for dental care in these settings, allowing patients to receive quality treatment without the need for complex machinery [20,21,22,23].

The effectiveness of ART has been extensively documented in numerous studies [24,25,26,27]. Research has shown that, when combined with materials like GIC, this technique not only provides durable restorations but also promotes a less traumatic treatment experience for children [28,29,30,31,32,33]. GIC, in addition to bonding well to the tooth structure, releases fluoride, helping to prevent further decay and improving long-term dental health [33,34,35,36,37]. Additionally, ART significantly reduces the pain and anxiety associated with traditional cavity removal with drills, fostering greater cooperation from pediatric patients [38,39,40,41].

In recent years, various studies have compared ART with other restorative techniques, such as the Hall Technique (HT) and Papacarie [22,42,43,44,45]. While the HT demonstrates higher success rates in terms of restoration longevity and retention, ART remains an effective treatment, especially when patient comfort and cost reduction are top priorities [2,46]. An interesting evolution of ART is the silver-modified ART technique, which involves the use of silver diamine fluoride (SDF) before restoration [47,48,49,50]. This combination has shown promising results, especially in high-risk caries situations, offering additional protection against further tooth deterioration [9,44,51,52,53,54].

**Figure 1:**
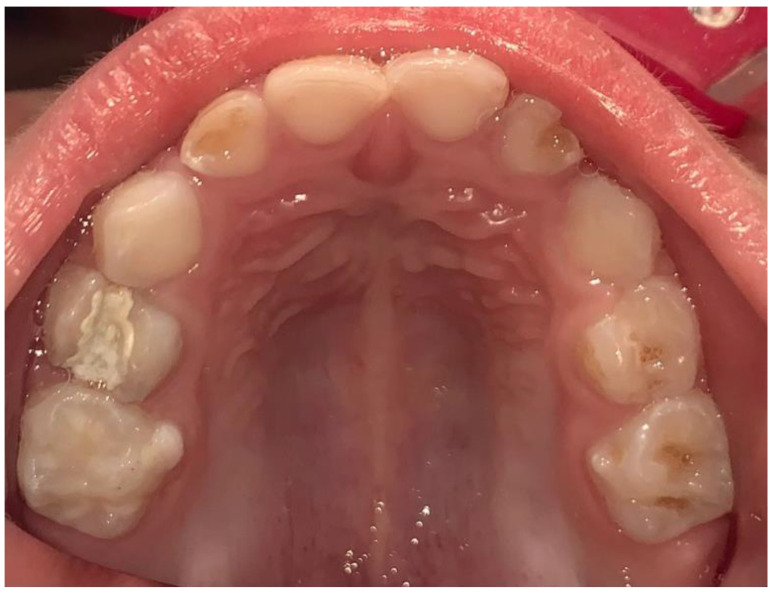
High-viscosity glass ionomer cement on 5.4.

The selection of materials plays a crucial role in the success of ART [44,55,56,57,58,59]. GIC, particularly high-viscosity glass ionomer cement (HV-GIC), have been identified as one of the best options for ensuring high performance in primary molars, reducing the risk of secondary caries and improving the longevity of restorations (Figure 1) [60,61,62,63,64,65]. Some studies have also suggested that small modifications to the technique, such as the inclusion of retentive features, could further enhance the durability of the fillings [42,43,45,66,67,68,69].

The aim of this discussion was to evaluate the effectiveness and potential of managing caries in primary teeth by analyzing ART’s clinical outcomes, the materials used, cost-effectiveness, and applicability in high-risk pediatric groups. We explored its comparison with other restorative techniques, such as the Hall Technique, Papacarie, and SMART, considering aspects like clinical success, treatment duration, and patient comfort. Additionally, we examined the performance of different materials—including GICs, compomers, and composites—focusing on their longevity, cost-effectiveness, and suitability for ART. The discussion also addressed the economic sustainability of ART, especially in low-resource settings, analyzing the balance between the initial costs and long-term benefits. Lastly, we assessed ART’s application in vulnerable pediatric populations, emphasizing its role in reducing the need for general anesthesia and specialized care. This analysis aimed to provide a comprehensive understanding of ART’s strengths and limitations in pediatric dentistry.

## 2. Materials and Methods

### 2.1. Protocol and Registration

The current systematic review followed the PRISMA guidelines (Preferred Reporting Items for Systematic Reviews and Meta-Analyses) and International Prospective Register of Systematic Review Registry procedures (full ID: 655547) [70,71].

### 2.2. Search Processing

The PubMed, Web of Science (WOS) and Scopus databases were examined from 2nd January 2015 to 31st January 2025 to search for articles published in the last 10 years (Table 1). The search strategy was created by combining terms relevant to this study’s purpose. In the advanced search string of the databases, the following keywords were applied using Boolean operators to combine terms pertinent to this study’s purpose: (“Dental Caries” OR “Caries” OR “Tooth Decay”) AND (“Atraumatic Restorative Treatment” OR “ART” OR “Minimally Invasive Treatment”) AND (“Child” OR “Children” OR “Pediatric Dentistry”).

### 2.3. Inclusion and Exclusion Criteria

The reviewers worked in groups to assess all relevant studies that evaluated or compared the effectiveness of restorative dental materials for the atraumatic treatment of caries in deciduous teeth using the following inclusion criteria:

- Open-access studies written in English;
- Studies conducted in vivo or on humans;
- Case-control studies, cohort studies, and randomized controlled trials (RCTs);
- Studies on ART for caries in deciduous teeth in children;
- Studies published in the last 10 years.

Studies that fulfilled at least one exclusion criterion were excluded: reviews, case reports and series, letters to the authors; animal models; studies in adults, and in vitro studies.

### 2.4. PICO Question

The PICO format is a framework used in qualitative research to structure clinical research questions. PICO addressed the question “Which dental restorative materials are most effective in the atraumatic treatment (e.g., ART—Atraumatic Restorative Treatment) of children with carious primary teeth in the context of pediatric dental care?” 

The PICO question was developed as follows:

- Population (P)—children with carious primary teeth;
- Intervention (I)—atraumatic treatment management (e.g., ART—Atraumatic Restorative Treatment);
- Comparison (C)—different dental restorative materials;
- Outcome (O)—effectiveness of the restorative materials in managing carious lesions in pediatric dental treatment.

### 2.5. Data Processing

Four independent reviewers (L.C., D.C., P.D.S, and P.N.) assessed the included studies’ quality using selection criteria, methods of outcome evaluation, and data analysis. The enhanced ‘risk of bias’ tool additionally provides quality standards for selection, performance, detection, reporting, and other biases. All differences were settled through conversation or collaboration with other researchers (G.D., C.M.N.M., A.P., A.D.I., and A.M.I.). The reviewers screened the records according to the inclusion and exclusion criteria. The 1202 selected articles were downloaded into “Zotero 6.0.36” for organization and analysis.

## 3. Results

### 3.1. Selection and Characteristics of the Study

**Figure 2:**
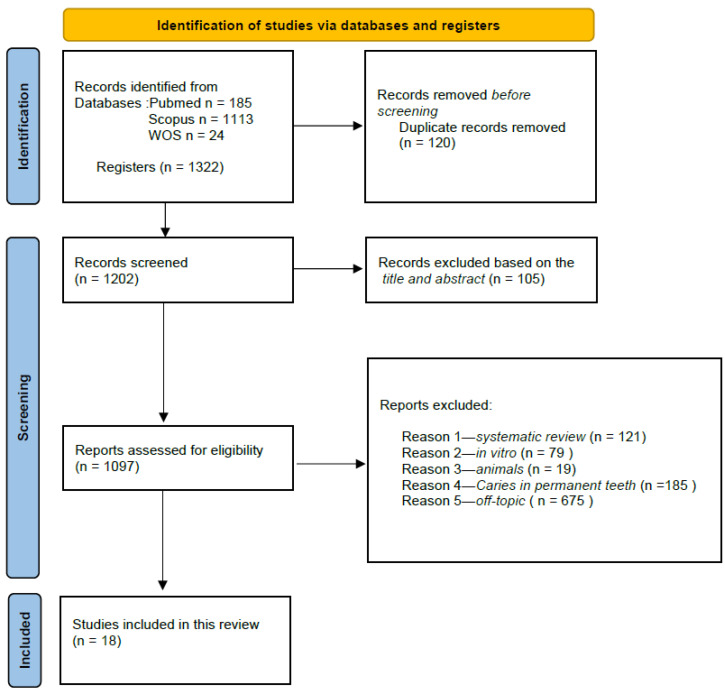
PRISMA flowchart.

This PRISMA (Preferred Reporting Items for Systematic Reviews and Meta-Analyses) diagram (Figure 2) illustrates a rigorous and systematic selection process to ensure that only relevant studies were included in the final review. A total of 1322 records were identified through electronic database searches, including PubMed (*n* = 185), Scopus (*n* = 1113), and WOS (*n* = 24). Records from other registers were not included. Before screening, 120 duplicate records were removed, reducing the number of records for screening to 1202. The most common reason for exclusion was studies being off topic (*n* = 675), followed by studies related to caries in permanent teeth (*n* = 185). Additionally, systematic reviews (*n* = 121), animal studies (*n* = 19), and in vitro studies (*n* = 79) were excluded to focus only on primary research relevant to the effectiveness of restorative dental materials for the atraumatic treatment of caries in deciduous teeth. Ultimately, only 18 studies met all inclusion criteria and were considered relevant for the final analysis. This selection process ensured the robustness and reliability of this systematic review. The selection process and summary of included records are illustrated in Figure 2, while the characteristics of the selected studies are presented in Table 2.

### 3.2. Quality Assessment and Risk of Bias of the Included Articles

The quality of the papers included was assessed by a reviewer, L.C., using the ROBINS-I (Risk of Bias in Non-Randomized Studies of Interventions), a tool developed to assess the risk of bias in the results of non-randomized studies that compare the health effects of two or more interventions. Seven points were evaluated, and each was assigned a degree of bias. A senior reviewer (F.I.) was consulted to clear up any doubts. The quality and risk of bias assessments included eighteen studies, as reported in Table 3. This evaluation assessed six domains (D1–D6) and provided an overall judgment for each study. The color-coded system distinguishes between a low risk of bias, indicated by green, and moderate risk, marked by yellow. No red indicators are present, suggesting that none of the included studies exhibit a high risk of bias. Examining the different domains, bias due to confounding (D1) is often rated as moderate risk, indicating that some studies may not have fully controlled for all factors capable of influencing the results. However, several studies show low risk in this area, demonstrating adequate management of potential confounders. Regarding participant selection (D2), a mix of low and moderate risk is observed, suggesting that while many studies adopted appropriate selection criteria, some may have non-optimal selection elements. The classification of interventions (D3) is mostly accurate, with many studies receiving a low risk rating. However, some show moderate risk, likely due to the less clear classification of the interventions used. Similarly, the risk due to deviations from intended interventions (D4) is generally low, indicating that, in most studies, deviations from planned interventions were minimal or well-controlled. A particularly positive aspect emerges in the handling of missing data (D5), where most studies are classified as low risk, suggesting that missing data were adequately managed or that the amount of missing information was negligible. Finally, the measurement of outcomes (D6) appears to be reliable in almost all cases, with predominantly green indicators confirming the consistency and validity of the measurements performed.

Overall, the table shows that most studies present a low risk of bias, making them methodologically sound and reliable for inclusion in systematic reviews. Some studies, with yellow indicators in more than one domain, show a moderate risk and should be interpreted with greater caution, especially concerning confounding factors and participant selection. However, the absence of red indicators suggests that none of the included studies have serious methodological limitations. Consequently, the data indicate a good level of methodological quality, with only a few areas requiring attention when interpreting the results

## 4. Discussion

ART has emerged as one of the most effective and minimally invasive treatment approaches for dental caries, particularly in pediatric dentistry [1,4,78,79]. The method, which relies on hand instruments to remove decayed tissue and uses adhesive restorative materials such as GIC, is often advocated for its simplicity, cost-effectiveness, and suitability in environments with limited access to advanced dental equipment [19,80,81]. Over the years, various studies have explored the efficacy of ART in primary molars, comparing it to other restorative methods and investigating the performance of different materials [31,48,82,83].

### 4.1. Comparison of ART with Other Restorative Techniques

One of the most comprehensive studies in this field was conducted by Hesse et al. (2016), who compared ART to the HT in the treatment of occluso-proximal cavities in primary molars [62]. This randomized clinical trial, involving 124 children, found that the HT provided significantly higher success rates regarding restoration survival and retention [62]. Additionally, children undergoing the HT experienced less discomfort and fewer behavioral challenges compared to those treated with ART. Hesse et al. (2016) concluded that while ART is effective, the HT might be preferable for certain clinical situations, particularly when patient comfort and longevity are prioritized [62].

Similarly Khalek et al. (2017) explored the discomfort levels and treatment times of ART versus Papacarie, an alternative caries removal technique that uses a solution to soften the carious tissue for easier removal [72]. This study demonstrated that Papacarie, although more time-consuming, resulted in less pain and discomfort for children compared to ART [72]. This is particularly relevant in pediatric dentistry, where patient comfort and the overall experience are crucial in gaining cooperation from young patients [72].

Furthermore, Mohammed et al. (2022) extended the evaluation of ART by investigating SMART technique, which includes the use of SDF prior to restoration [76]. The study found that SMART, which combines the caries-arresting effects of SDF with the restorative capabilities of ART, showed higher success rates for caries prevention and restoration longevity [76]. 

A study comparing the clinical efficacy and cost-effectiveness of ART with SMART for occlusal restorations in primary molars was carried out by Aly et al. (2023) [56]. 

The findings showed no appreciable variations in survival rates between the two methods. However, SMART was found to be more economical, with a shorter treatment time [56]. Clinical results from both methods were similar, including minimal adaptation and no secondary caries [56]. The study found that SMART is a cost-effective and time-efficient substitute for ART that can be used in childhood for minimally invasive dentistry [56]. These findings emphasize that while ART is effective, combining it with adjuncts like SDF can yield even better results, particularly in high-risk caries situations [56,76].

Additionally, a preliminary study by de Souza et al. (2022) examined the combination of ART with Brix3000™ papain gel for caries removal in primary teeth [44]. While this approach required a longer treatment time compared to ART alone, it was equally well-accepted by children and did not result in increased discomfort [44]. These findings suggest that integrating enzymatic methods like Brix3000™ into ART could offer a viable alternative for minimally invasive caries management, although further research is needed to confirm its long-term benefits [44].

### 4.2. Materials Used in ART Restorations

The materials used in ART restorations play a critical role in the success and longevity of the procedure. de Medeiros Serpa et al. (2017) conducted a comparative study assessing the effectiveness of GIC versus composite resin (CR) in ART restorations [55]. The study found that while both materials effectively treated carious lesions, CR exhibited better wear resistance and marginal integrity over time [55]. However, GIC is often preferred due to its lower cost and easier application, making it more suitable for the ART technique, particularly in settings with limited resources [55].

**Figure 3:**
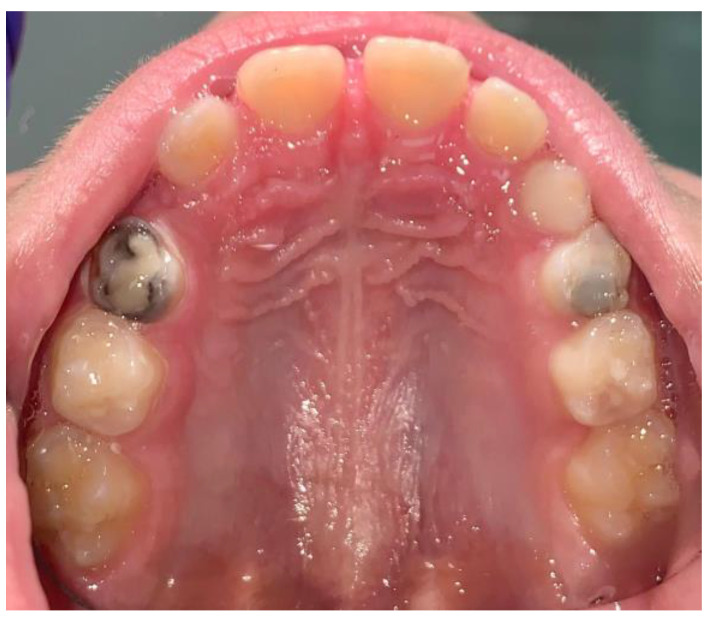
SMART 5.4 and 6.4.

Olegário et al. (2017) focused on the performance of different GICs in ART restorations, particularly analyzing their long-term success rates [73]. In the study, they compared GC Gold Label 9, Vitro Molar, and Maxxion R, finding that GC Gold Label 9 provided the highest success rates over a one-year follow-up period due to its superior adhesion and durability [73]. The results underscored the significance of selecting high-quality ART materials to ensure optimal restoration performance [73]. An important consideration emerging from the study by Meng Jiang et al. (2020) is that the prior application of SDF can significantly enhance the therapeutic approach for caries in primary teeth [60]. This study demonstrated that although the success rates of restorations did not differ significantly between groups treated with and without SDF, the use of SDF reduced the time required for restoration placement [60,82,84,85,86]. Therefore, the application of SDF presents itself as an advantageous option, particularly for young and reluctant children, as it shortens the procedure duration without compromising ART success (Figure 3) [60,87].

De França Lopes et al. (2018) also contributed to this field by comparing glass carbomer cement and HV-GIC [57]. Their results indicated that GIC performed better in terms of success rates over 12 months, reaffirming that HV-GIC is a reliable choice for ART restorations in primary molars [45,57,88].

Further studies by Olegário et al. (2019) and Pássaro et al. (2022) continued to explore the longevity of different materials in ART [32,74]. They compared GIC, compomer, and glass carbomer, finding that GIC outperformed both compomer and glass carbomer in terms of survival rates, particularly in high-stress occlusal areas [32,74]. This highlights the importance of material selection, especially when considering the occlusal forces and wear patterns typically seen in primary molars [32,74].

Hesse et al. (2016) investigated the longevity of approximal ART restorations in primary molars using different insertion techniques and surface protection materials [64,89,90]. They found that the bilayer technique improved restoration survival compared to the conventional method [64,91,92,93,94,95]. Additionally, applying a nano-filled coating increased the durability of conventional restorations [41,42,43,64,96,97,98]. The most common cause of failure was bulk fracture, while pulp inflammation was less frequent and equally distributed among groups [30,64,66,67,99]. No clinical factors influenced restoration survival [64]. The results suggest that combining the bilayer technique and a nano-filled coating can enhance the success of approximal ART restorations in primary teeth [52,64,100,101].

### 4.3. Cost-Effectiveness of ART

In addition to clinical outcomes, the cost-effectiveness of ART has been a focal point of several studies, particularly in low-resource settings. Olegário et al. (2020) explored the economic viability of different GICs, including Fuji IX, Vitro Molar, and Maxxion R, in ART restorations [75,102]. They found that while Fuji IX had a higher initial cost, its superior longevity made it the most cost-effective option in the long run [39,75,103,104,105]. This study provided compelling evidence that investing in high-quality materials can ultimately reduce the frequency of restoration failures and the need for retreatment, thereby minimizing overall treatment costs [75,106,107,108].

Similarly, Garbim et al. (2024) compared the cost-effectiveness of two encapsulated GICs: Riva Self Cure and Equia Forte [77,109,110,111]. The study showed that while both materials demonstrated similar clinical results, Riva Self Cure was more affordable and still provided reliable results, making it an ideal choice for public health initiatives where budget constraints are a significant concern [77,112,113,114]. This finding suggests that ART’s affordability can be maintained without compromising treatment outcomes, offering an economically viable solution for both private and public dental practices [77].

The long-term success of ART restorations has been widely studied, with several studies providing valuable insights into their durability. Faustino-Silva et al. (2019) conducted a four-year longitudinal study comparing two different GICs, Ketac Molar Easymix^®^ (Seefeld, Germany) and Vitro Molar^®^ (Rio de Janeiro, Brasil), in ART restorations [63]. They found that both materials provided excellent clinical outcomes, with Ketac Molar exhibiting slightly better retention and fewer signs of wear after four years. This study highlighted ART’s ability to provide lasting restorations, even in high-risk pediatric populations [63,115,116,117,118].

Pesaressi et al. (2024) examined the use of retentive grooves in ART restorations and found that incorporating these grooves significantly improved the survival rate of restorations over 12 months [58]. Restorations with retentive grooves were less likely to fail due to bulk fractures, a common mode of failure in ART [58,119,120,121]. This study suggests that small modifications to the ART technique, such as the addition of retentive features, can enhance the overall longevity and performance of restorations [58,122,123,124].

### 4.4. The Effectiveness of ART in High-Risk Pediatric Groups

ART has also proven to be particularly effective in managing caries in special populations, such as children from underserved communities. Arrow et al. (2018) explored the effectiveness of ART in rural Aboriginal communities in Western Australia, finding that ART was an invaluable tool for treating early childhood caries [59,125]. The study by Arrow et al. (2018) highlights the significant potential of ART as a primary dental care strategy for young children, particularly in rural and remote settings. ART demonstrated strong clinical effectiveness, reducing the need for general anesthesia by 44% while also improving children’s oral health-related quality of life [59,126,127]. It was well-tolerated, cost-effective, and suitable for low-resource environments. As part of a holistic care model including preventive measures and community involvement, ART offers a scalable and sustainable solution to early childhood caries in underserved populations [59,128,129].

**Figure 4:**
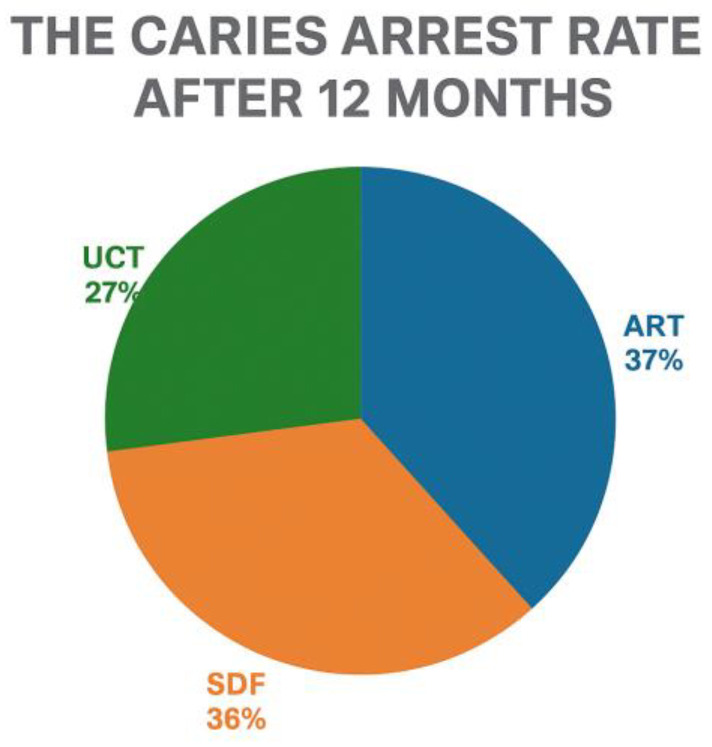
After 12 months, ART demonstrated a caries arrest rate of 87.2%, which was slightly higher than SDF (84.6%) and significantly better than UCT (61.6%).

Furthermore, Hamza et al. (2024) compared ART with other minimally invasive techniques, such as SDF and UCT in preschool-aged children [61,130]. After 12 months, ART demonstrated a caries arrest rate of 87.2%, slightly higher than SDF (84.6%) and significantly better than UCT (61.6%). While all three treatments showed 100% success at the 3-month follow-up, differences emerged over time, highlighting ART’s superior long-term efficacy compared to UCT (Figure 4). They found that ART had a significantly higher caries arrest rate compared to UCT, though it required a longer treatment time [61,118,131]. In terms of treatment logistics, ART required a longer mean treatment time (7.9 min) than SDF (3.4 min) and UCT (4.1 min). Children treated with ART also showed slightly higher levels of anxiety post-treatment, likely due to the use of instruments during the procedure. However, there were no significant differences in adverse events or parental aesthetic concerns between the groups [61,132,133].

Several clinical studies have evaluated the effectiveness and clinical outcomes of ART in pediatric dentistry. Hesse et al. (2016) reported a 52.8% survival rate for approximal ART restorations after three years, highlighting both the bilayer technique and the application of a nano-filled coating significantly improved restoration longevity compared to conventional approaches [64]. Similarly, Faustino-Silva et al. (2019) demonstrated excellent long-term outcomes in children aged 18 to 36 months, with ART restorations showing a 94% success rate at one year, 87.5% at two years, and 82.9% at four years [63].

In another randomized clinical trial, de França Lopes et al. (2018) found that Class II ART restorations using HV-GICs had a survival rate of 83% at six months, which remained high (86%) after twelve months, outperforming glass carbomer materials [57].

Olegário et al. (2019) observed three-year survival rates of 83% for GIC, 78% for compomer, and 62% for glass carbomer in occlusal ART restorations. For occlusal-proximal restorations, GIC and compomer both achieved 56% survival, while glass carbomer performed less favorably (36%) [73]. Regarding patient comfort, Abdul Khalek et al. (2017) reported that ART was associated with moderate discomfort but was better tolerated than conventional rotary instrumentation. Additionally, Papacarie was associated with significantly lower pain levels, although it required a longer application time [72].

Jiang et al. (2020) found that ART restorations in primary teeth achieved comparable 24-month success rates regardless of prior treatment with SDF, with Class I lesions performing best (50% success), while multi-surface restorations showed significantly lower rates [60]. Mohammed et al. (2022) observed higher clinical success of the SMART technique (ART + SDF) compared to ART alone at 6 and 12 months, suggesting that the addition of SDF may enhance outcomes [76]. Aly et al. (2023) confirmed these findings, reporting similar survival times (12 months) for both SMART and ART, with SMART being more cost-effective and requiring significantly less chair time [56]. Regarding material selection, Olegário et al. (2019) found that high-viscosity GIC (Fuji IX) had a significantly higher two-year survival rate (72.7%) compared to lower-cost alternatives [74].

Pássaro et al. (2022) evaluated Giomer resin composites versus GICs in occluso-proximal ART restorations and found no significant difference in survival after 24 months [32].

Additionally, studies by Garbim et al. (2024) and Pesaressi et al. (2024) highlighted the moderate survival of encapsulated GICs in Class II restorations, with Pesaressi et al. noting that incorporating retentive grooves significantly improved outcomes (from 77.2% to 91.8% at 12 months) [58,77]. Lastly, de Souza et al. (2022) confirmed that ART is a well-tolerated procedure among children, with no significant differences in pain or acceptability whether it is performed alone or in combination with Brix3000™, a papain-based gel. Collectively, these findings reinforce ART as a clinically effective, child-friendly, and economically viable approach for treating dental caries in primary teeth, particularly when enhanced by appropriate materials and techniques [44].

## 5. Conclusions

In conclusion, ART has demonstrated consistent effectiveness as a minimally invasive method for treating dental caries, especially in pediatric populations. Its success hinges on the careful selection of restorative materials, such as GICs, which are known for their longevity and ability to bond effectively to the tooth structure. Studies have shown that combining ART with adjunct techniques like the HT or Papacarie enhances its efficacy, particularly in complex or challenging cases. ART’s cost-effectiveness is one of its most significant advantages, particularly in low-resource settings, where it provides an accessible, budget-friendly treatment option. It is particularly valuable in underserved and rural communities, where access to advanced dental care may be limited. Furthermore, ART’s long-term success, especially with the use of high-quality GICs, solidifies its role as a reliable and versatile treatment option for managing caries in primary molars. The simplicity of ART, along with its focus on reducing patient discomfort, leads to high acceptance rates, especially among children. This results in improved cooperation and better clinical outcomes, making ART an invaluable tool in pediatric dentistry.

## Figures and Tables

**Table 1:** Indicators for database searches.

Article screening strategy	Keywords: “Dental Caries, Caries, Tooth Decay, Atraumatic Restorative Treatment, ART, Minimally Invasive Treatment, Child, Children, Pediatric dentistry”
	Boolean Indicators: OR and AND
	Timespan: 2nd January 2015 to 31st January 2025
	Electronic databases: PubMed, Scopus, WOS.

**Table 2:** Analysis of the studies included in the Discussion section from 2015 to 2024.

Authors	Type of Study	Patients	Aim of the Study	Materials and Methods	Conclusions
Hesse et al., 2016 [64]	RCT	208 children (6–7 years)	Compare GIC insertion techniques and surface protection	Four groups were studied, conventional or bilayer GIC with petroleum jelly or a nano-filled coating, with a follow-up period of 36 months.	The bilayer technique and nano-filled coating helped the restorations last longer.
Hesse et al., 2016 [62]	RCT	124 children (6–8 years)	Compare ART and the HT for treating occluso-proximal cavities in primary molars	Two groups were compared: ART with GIC and HT with metal crowns.	ART is more effective and acceptable for managing occluso-proximal caries in primary molars, especially in low-resource settings, to support clinical and public health decision-making.
Abdul Khalek et al., 2017 [72]	RCT	50 children (4–8 years)	Compare pain and discomfort during caries removal using Papacarie vs. ART	Two groups were studied: (1) Papacarie gel for removing cavities and (2) ART with hand tools. Pain was measured using the SEM scale (Sound, Eye, and Motor scale).	Papacarie caused less pain and discomfort than ART, but took a bit more time. It is a more comfortable option for young or anxious children.
de Medeiros Serpa et al., 2017 [55]	Randomized, split-mouth, blind clinical trial	86 children (4–8 years), 216 teeth	To evaluate the clinical and radiographic success of ART using GIC and CR in primary molars	A total of 108 restorations with GIC (Ketac Molar Easy Mix) and 108 with CR (Filtek Z250) were studied.	ART with both materials worked well. CR had better wear resistance, while GIC had a higher risk of secondary cavities. ART is a good option for children’s dental treatments.
Olegário et al., 2017 [73]	RCT	150 children (4–8 years)	To evaluate the survival rate of occlusal ART restorations in primary molars using three different GICs	Three groups were studied: GC Gold Label 9, Vitro Molar, and Maxxion R.	Low-cost GICs (Vitro Molar and Maxxion R) lasted less than GC Gold Label 9, showing that they perform worse in the long term.
de França Lopes et al. (2018) [57]	RCT	33 children (6–10 years)	Compare the survival rates of ART Class II restorations using glass carbomer and HV-GIC	A total of 59 restorations were placed by a pediatric dentist; two calibrated, blinded examiners assessed restorations at 6 and 12 months.	HV-GIC had much higher success rates than glass carbomer, which showed more wear and edge problems. HV-GIC is a better choice for ART Class II restorations.
Arrow et al., 2020 [59]	RCT	26 communities; preschool children (0–4 years)	Evaluate an ART-based primary care model for managing early childhood caries in Aboriginal children	The study had two groups: immediate vs. delayed treatment. Participants were checked at the start and after 12 months, with support from Aboriginal research assistants.	The ART approach reduced the need for specialist visits and general anesthesia, improved oral health, and saved costs.
Faustino-Silva et al., 2019 [63]	Randomized, double-blind clinical trial	25 children (18–36 months)	Evaluate ART for ECC (Early Childhood Caries) over 4 years.	A total of 25 children (18–36 months) with 100 decayed molars received ART with two types of cements.	ART was effective, with similar performance for both GICs.
Olegário et al., 2019 [74]	RCT	568 children (4–7 years old)	Compare ART with GIC, COM, and CAR	A total of 568 children (287 occluso-proximal, 281 occlusal cavities) were randomly assigned to GIC, COM, or CAR; follow-up was conducted at 2, 6, 12, 18, 24, and 36 months.	GIC and COM had better survival than CAR. CAR is not recommended for ART.
Meng Jiang et al., 2020 [60]	RCT	194 preschool children (3–4 years old)	Test SDF’s effect on ART success	Cavitated caries lesions were treated with either 38% SDF or a placebo (tonic water), followed by ART restoration after 10 weeks. Success rates were evaluated over 24 months.	SDF reduced the treatment time and improved the cooperation of young children.
Olegário et al., 2020 [75]	RCT	150 children (4–8 years)	Evaluate the survival and cost-effectiveness of 3 GICs in ART restorations	Fuji IX, Vitro Molar, Maxxion R were compared over 2 years for survival and costs.	Fuji IX showed highest survival (72.7%) and cost-effectiveness over 2 years.
Mohammed et al., 2022 [76]	RCT	30 children (3–6 years)	Compare SMART (Silver-Modified Atraumatic Restorative Treatment) and ART outcomes	Split-mouth design with GIC and SDF treatments	SMART had higher success rates than ART.
de Souza et al., 2022 [44]	RCT	20 children (3–9 years)	Compare ART with/without Brix3000™ for time, pain, and acceptability Test Brix3000™ in ART	Measured time, pain, and acceptability (hedonic scale) during caries removal	Brix3000™ took more time but did not affect pain or acceptance.
Pássaro et al., 2022 [32]	RCT	182 children (4–8 years)	Compare GIC vs. GCR (Giomer Composite Resin) in ART	GIC vs. GCR in molar restorations, 24-month follow-up	GCR had more failures than GIC.
Aly et al., 2023 [56]	RCT	67 children (5–9 years)	Compare SMART vs. ART	SMART and ART, 12-month follow-up, cost and performance analyses	Both methods were effective, but SMART was quicker and cheaper.
Garbim et al., 2024 [77]	RCT	152 children (4–8 years)	Compare GIC survival with EF (EQUIA Fil) and RSC (Riva Self Cure)	ART restorations with EF or RSC, evaluated over 24 months	RSC matched EF’s success and was more cost-effective.
Pesaressi et al., 2024 [58]	RCT	187 children (3–7 years)	Evaluate Class II ART restoration survival with/without grooves.	293 restorations with GIC; survival was assessed at 6 and 12 months	Grooves improved success, especially in high-caries cases.
Hamza et al., 2024 [61]	RCT	135 children (3–5 years)	Evaluate ART, SDF, and UCT (Ultraconservative Treatment) in arresting dentin caries	Three groups were compared; follow-ups were conducted at 3, 6, and 12 months; caries arrest, treatment time, and anxiety were measured.	ART and SDF had the best success. SDF took less time and caused less anxiety.

**Table 3:** A tabular summary of the risk of bias assessment of 18 studies, evaluated across six domains.

Authors and Year	D1	D2	D3	D4	D5	D6	Overall
Hesse et al., 2016 [64]	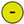	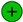	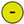	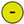	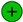	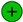	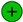
Hesse et al., 2016 [62]	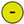	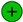	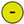	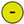	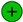	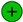	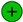
Abdul Khalek et al., 2017 [72]	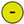	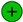	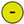	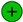	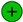	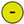	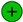
de Medeiros Serpa et al., 2017 [55]	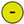	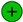	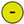	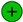	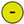	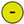	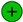
Olegário et al., 2017 [73]	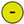	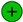	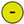	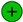	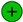	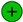	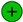
de França Lopes et al., 2018 [57]	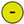	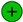	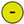	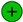	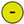	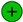	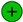
Arrow et al., 2020 [59]	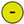	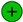	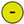	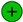	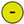	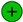	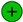
Faustino-Silva et al., 2019 [63]	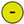	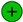	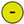	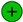	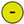	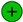	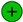
Olegário et al., 2019 [74]	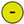	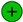	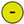	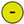	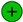	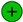	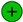
Meng Jiang et al., 2020 [60]	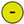	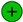	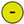	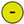	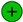	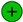	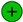
Isabel Cristina Olegário et al., 2020 [75]	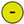	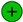	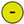	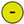	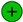	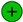	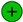
Mohammed, S.M.E et al., 2022 [76]	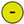	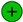	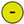	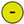	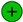	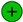	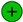
T. F. de Souza et al., 2022 [44]	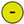	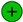	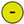	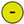	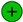	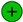	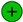
Pássaro et al., 2022 [32]	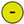	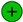	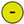	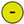	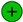	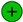	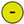
Abla Ahmed Mohamed Aly et al., 2023 [56]	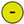	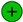	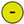	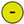	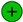	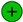	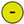
Jonathan Rafael Garbim et al., 2024 [77]	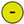	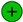	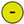	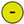	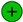	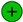	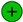
E. Pesaressi et al., 2024 [58]	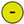	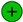	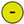	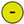	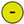	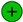	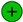
Basma Elsayed Hamza et al., 2024 [61]	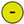	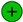	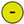	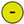	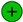	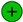	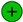
**Domains**					**Judgement**		
**D1: Bias due to confounding**					Very High		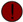
D2: Bias arising from the measurement of the exposure.					High		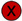
D3: Bias in the selection of participants in the study (or in the analysis).					Some Concerns		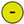
D4: Bias due to post-exposure interventions.					Low		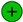
D5: Bias due to missing data.					No Information		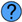
D6: Bias arising from the measurement of the outcome.							

## Data Availability

The data are contained within the article.

## Abbreviations

Abbreviation used in this review.

**Abbreviation**	**Definition**
ART	Atraumatic Restorative Treatment
CAR	Glass Carbomer
COM	Compomer
CR	Composite Resin
ECC	Early Childhood Caries
EF	EQUIA Fil
GCR	Giomer Composite Resin
GIC	Glass Ionomer Cement
HT	Hall Technique
HV-GIC	High Viscosity Glass Ionomer Cement
RCT	Randomized Clinical Trial
RSC	Riva Self Cure
SDF	Silver Diamine Fluoride
SEM scale	Sound Eye and Motor scale
SMART	Silver-Modified Atraumatic Restorative Treatment
UCT	Ultraconservative Treatment
